# The Inner Ear and Aging Brain: A Cross-Sectional Study of Vestibular Function and Morphometric Variations in the Entorhinal and Trans-Entorhinal Cortex

**DOI:** 10.1007/s10162-025-00977-2

**Published:** 2025-02-24

**Authors:** Claire J. Vania, Dominic Padova, J. Tilak Ratnanather, Yuri Agrawal

**Affiliations:** 1 Department of Biomedical Engineering, Whiting School of Engineering, Johns Hopkins University, Baltimore, MD USA; 2 Center for Imaging Science and Department of Otolaryngology-Head and Neck Surgery, Johns Hopkins University School of Medicine, Baltimore, MD USA; 3 Center for Imaging Science and Institute for Computational Medicine, Department of Biomedical Engineering, Johns Hopkins University, Baltimore, MD USA; 4 Department of Otolaryngology-Head and Neck Surgery, Johns Hopkins University School of Medicine, Baltimore, MD USA; 5 Department of Otolaryngology-Head and Neck Surgery, University of Colorado, Anschutz Medical Campus, Aurora, CO USA

**Keywords:** Vestibular function, Aging, Entorhinal cortex, Trans-entorhinal cortex, Shape analysis, MRI

## Abstract

**Purpose:**

While the vestibular system is crucial for balance, posture, and stable vision, emerging evidence connects vestibular loss in older adults to spatial cognitive deficits. However, the specific neural pathways remain unclear. This study examines morphometric changes in the entorhinal cortex (ERC) and trans-entorhinal cortex (TEC), key regions in the vestibular spatial cognitive network, with vestibular function.

**Methods:**

This cross-sectional study used T1-weighted MRI images and vestibular physiological data from 103 Baltimore Longitudinal Study of Aging participants (74 males and 29 females). Vestibular function was assessed through the cervical vestibular-evoked myogenic potential (cVEMP), ocular VEMP (oVEMP), and video head–impulse test (vHIT), examining both categorical presence/absence of responses and continuous measures (cVEMP amplitude, oVEMP amplitude, and VOR gain). Morphometric changes in the ERC and TEC were analyzed by examining surface expansions and contractions relative to average shapes.

**Results:**

Reduced saccular function correlated with surface expansion in the left ERC’s pro-rhinal, right ERC’s intermediate caudal and superior regions, and right TEC’s sulcal region. The decreased utricular function was associated with surface contraction in the left lateral TEC, left ERC’s anterior sulcal and trans-entorhinal regions, and surface expansion in the lateral region of the left ERC. Reduced canal function showed surface contraction in the right ERC’s pro-rhinal and lateral regions and the right TEC’s posterior sulcal and trans-entorhinal regions.

**Conclusion:**

These findings highlight the intricate link between vestibular function and ERC/TEC morphology, emphasizing their role in spatial and cognitive abilities. Future research will assess if structural changes due to vestibular loss contribute to cognitive deficits in aging and Alzheimer’s disease.

**Supplementary Information:**

The online version contains supplementary material available at 10.1007/s10162-025-00977-2.

## Introduction

The vestibular system has evolved to sense the motions that arise from head movements and the inertial effects of gravity. Although the vestibular system is widely known for its role in balance, posture, and stable gaze, mounting research demonstrates its importance in cognition and aging [1–6]. Several recent studies have found that vestibular impairment is significantly associated with cognitive deficits, such as the decline in visuospatial functions in older adults, spatial memory, and navigation [7–10]. However, the exact central nervous system pathways through which the loss of peripheral vestibular function affects cognition in older adults remain poorly defined. Several neuroimaging studies of vestibular function have found structural changes in the vestibular spatial cognitive network, which includes the hippocampus, thalamus, basal ganglia, entorhinal cortex (ERC), and trans-entorhinal cortex (TEC) [11, 12]. In a study of 97 healthy, older adults, Padova et al. (2021) found that reduced saccular function was associated with reduced gray matter volumes bilaterally of the basal ganglia and thalamus and that reduced horizontal canal function was associated with reduced gray matter volume in the left hippocampus [13].

Entorhinal cortex (ERC) and trans-entorhinal cortex (TEC) have been hypothesized to be part of vestibular pathways involved in transmitting vestibular information to vestibular cortex [12]. Beyond their involvement in vestibular pathways, studies have linked ERC and TEC to memory processing, spatial memory and navigation, and cognition [14–18]. Despite the importance of the ERC and TEC in vestibular and cognitive functions, few studies have linked vestibular function to ERC structure in humans. In a cross-sectional study of 80 healthy older adults, Jacob et al. (2020) found that reduced saccular function was significantly associated with a reduction in ERC volume and ERC compression [19]. Notably, it has been the only study up to date to look at saccular function–ERC structure relationships in depth. The dearth of ERC and TEC findings could be a consequence of a lack of power due to small sample sizes or inadequate brain mapping methods. The whole-brain voxel-based and volume-based methods that dominate the neuroimaging analysis of vestibular function have known problems with detecting small, non-focal, or non-uniformly distributed effects. Region-of-interest–based shape analysis has emerged to sensitively track structural alterations that such whole-brain voxel–based and volume-based brain mapping methods may miss.

This study investigates the relationship between age-related vestibular end-organ loss and the morphology of the ERC and TEC while adjusting for sex, age, and intracranial volume in 103 older adults from the BLSA using vestibular data and MRI images. This study extends a previous study of the same cohort [19] to report new relationships between end-organ function and morphology in the ERC and TEC with new anatomical interpretations. We hypothesized that reduced vestibular function would be associated with surface compressions in the subregions of the ERC and TEC that subserve spatial cognitive ability.

## Methods and Material

### Data

To better understand how community-dwelling adults age, the Baltimore Longitudinal Study of Aging (BLSA) was established in 1958 [20]. We selected a subset of the research participants who underwent brain MRI scans and vestibular testing in the same study visit between 2013 and 2015 and were $\ge$ 60 years old. From the total participant cohort, 103 individuals matched the criteria for our study subset. The group’s mean age (± SD) was 76.99 years (± 8.7), and 72% were males. All participants provided written informed consent and were cognitively healthy and diagnosed with no history of vestibular, ophthalmological, microvascular, or neurodegenerative disease. The BLSA study protocol (03-AG-0325) was approved by the National Institute of Environmental Health Sciences Institutional Review Board.

### MRI Testing

The National Institute on Aging’s (NIA) Clinical Research Unit used a 3 T Philips Achieva scanner to conduct the MRI scans. Sequences included a T1-weighted volumetric scan magnetization prepared rapid acquisition with gradient echo (MPRAGE; TR = 6.5 ms, TE 3.1 ms, flip angle = 8°, 256 × 256 image matrix, 170 slices, voxel size = 1.0 × 1.0 mm, slice thickness = 1.2 mm, FOV = 256 × 240 mm). T1-weighted volumetric images were collected in the sagittal plane.

### Vestibular Testing

Vestibular function testing includes the cVEMP test to assess saccular function, the oVEMP test to assess utricular function, and the video head impulse test (vHIT) which measures VOR gain to assess semicircular canal functioning. A vestibular-evoked myogenic potential (VEMP) is a short-latency potential that is evoked by activating vestibular receptors with sound or vibration. It is produced by electromyographic impulses that have been modified, either from the inferior oblique muscle for the ocular VEMP (oVEMP) or the sternocleidomastoid muscle for the cervical VEMP (cVEMP). The otolith organs appear to be the source of these reflexes and hence can be used to test the function of otolith organs [21, 22]. A commercial electromyographic system (software version 14.1; Carefusion Synergy, Dublin, OH, USA) was used for recording the VEMP signal. Electromyogram signals were recorded with disposable, self-adhesive, pre-gelled Ag/AgCl electrodes with 40-inch safety lead wires from GN Otometrics (Schaumburg, IL, USA). Electromyogram signals were amplified 2500 × and band-pass filtered, 20–2000 Hz for cervical vestibular-evoked myogenic potentials and 3–500 Hz for ocular vestibular-evoked myogenic potentials.

Both continuous and categorical measures were utilized for cVEMP, oVEMP, and VOR gain as independent variables of interest in separate analyses. For cVEMP and oVEMP, the continuous measure was the higher amplitude from either ear (corrected in the cVEMP case), and the categorical measure was a bilaterally absent response versus any present response. For VOR gain, continuous measures represented the average VOR gain from both eyes, whereas categorical measures classified responses as impaired (gain < 0.8) or unimpaired.

#### Cervical VEMP (cVEMP)

Participants laid with upper bodies increased at 30° from horizontal. A non-inverting electrode was placed at the mid-point of the sternocleidomastoid muscle, an inverting electrode was placed on the sternoclavicular junction, and a ground electrode was placed on the manubrium sterni. During stimulation and recording, participants were instructed to lift their heads from the headrest to provide tonic background sternocleidomastoid activity. A pre-stimulus rectified surface electromyogram signal of at least 30 µV was used required for accepting a cervical vestibular–evoked myogenic potential tracing [22]. Bursts of monoaural, 500 Hz, 125 dB sound stimuli were delivered using headphones (VIASYS Healthcare, Madison, WI, USA). The background EMG activity was obtained 10 ms before the start of the sound stimulus, and it was used to normalize the recorded myogenic potentials. In the analyses, the greater cVEMP from either ear was used. In accordance with established rules, an absent response was defined as one that fell below a threshold level. The assessment was then repeated to ensure accuracy [14, 16].

#### Ocular VEMP (oVEMP)

For ocular vestibular-evoked myogenic potential testing, participants laid with upper bodies increased at 30° from horizontal. A non-inverting electrode was placed on the cheek ≈3 mm below the eye, directly beneath the pupil, an inverting electrode was placed 2 cm below the non-inverting electrode, and a ground electrode was placed on the manubrium sterni. Before stimulation, participants were instructed to perform 20° vertical 19 saccades to ensure that symmetrical signals were recorded from both eyes. If signals showed > 25% asymmetry, the electrodes were removed, and new ones were applied. Participants were instructed to maintain a 20° up gaze during ocular vestibular-evoked myogenic potential stimulation and recording [22]. The midline of the face was tapped with a reflex hammer at the hairline and about one-third of the space between the inion and nasion. In this investigation, the best response from either ear was used. An absent answer was recorded and repeated for confirmation if the response fell below the predetermined threshold levels [21, 23].

#### VOR Gain Testing

The horizontal vestibular ocular reflex (VOR) was assessed using the video head impulse test (vHIT). The vHIT was performed in the plane of the right and left horizontal semicircular canals using the EyeSeeCam system (Interacoustics, Eden Prairie, MN, USA). The patient’s head was pitched down 30° to place the horizontal canals in the plane of stimulation, and subjects were asked to fix their gaze on a wall target about 1.5 m away. The head was moved at a small amplitude (approximately 5–15°) with high velocity (typically 150–250°/s) horizontally toward the right and left side at least 10 times in both directions. The direction of the head movement was randomized to be unpredictable. The EyeSeeCam system measured eye velocity and head velocity, and a corresponding VOR gain was calculated by dividing the eye velocity by the head velocity. A normal, compensatory VOR gain should equal 1.0. VOR gains less than 0.8, along with compensatory refixation saccades, suggesting a loss of peripheral vestibular function [23].

### Pipeline

**Fig. 1:**
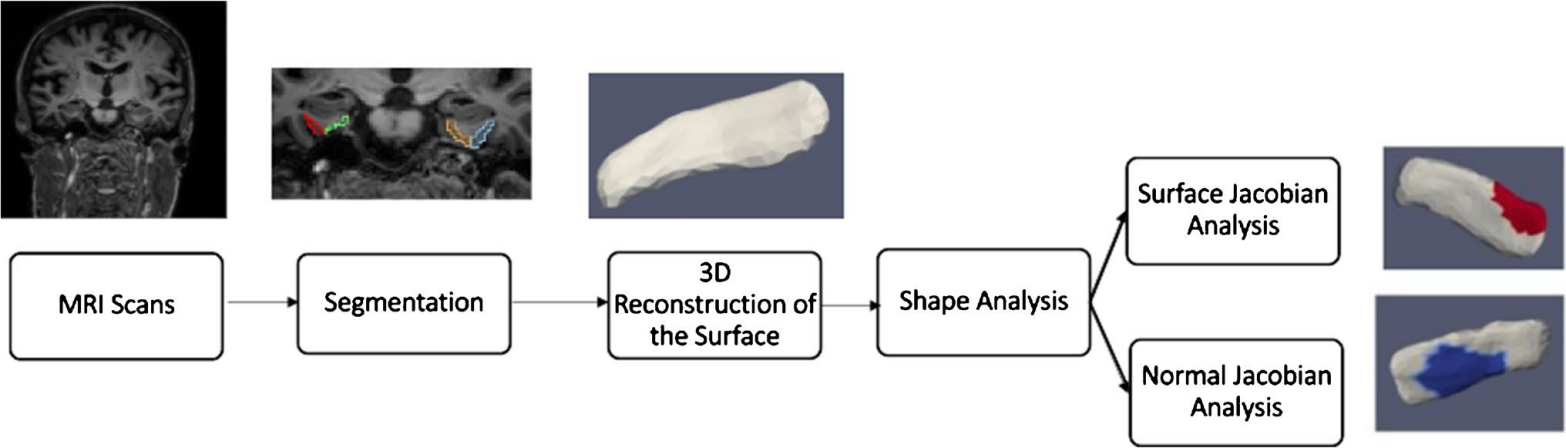
Pipeline flowchart

The general outline for shape analysis is shown in Fig. 1. A binary segmentation volume is created by segmenting the structure of interest from the brain’s MRI scans. A 3D surface is then obtained by triangulating the binary volume. Shape descriptors were determined by measuring how far each subject’s shape deviated from the population template. Regression is employed in statistical analysis, with shape descriptors as the dependent variable and vestibular factors as the independent variables. The following subsections go into further detail about each phase.

#### Segmentation and 3D Reconstruction of Surfaces

T1 scans were automatically segmented by registering them to multiple atlases using large deformation diffeomorphic metric mapping (LDDMM) [24]. The parcellation of the scans was based on the multiple-atlas likelihood fusion (MALF) algorithm [25, 26], with 286 defined structures. The parcellation was done through MRICloud, an online neuroinformatics platform that provides tools for automatic brain parcellation and surface registration (https://www.mricloud.org/). Surfaces were created from the binary segmentations using restricted Delaunay triangulation [27–29]. Since the accuracy of the segmentations and the surface reconstruction were crucial to the outcomes, quality control was carried out at every step, and manual editing of segmentations and surface meshes was done when necessary.

#### Shape Analysis

For shape analysis, the population of surface meshes was rigidly aligned and used to create a “mean shape” called the template. Templates were made separately for each structure’s left and right sides. The generated template is label- and group-blind and serves as a coordinate system for the population average. Each subject surface was registered to this template, first rigidly and then using surface LDDMM. The algorithm computes a smooth invertible mapping of the triangulated surface template onto the target surfaces. MRICloud provides the public with access to this pipeline [30, 31]. The MRICloud diffeomorphic registration pipeline yields two deformation values per vertex: Jacobian determinant of the 3D surface transformation and the surface Jacobian. The Jacobian determinant is a scalar that describes the volume change due to the diffeomorphic change of coordinates. The surface Jacobian is a scalar that describes the ratio between the surface area of the faces attached to a vertex before the transformation (i.e., the surface template) and following it (i.e., the surface template mapped to the target surface). Because surface shape can vary independently in terms of expansion/compression tangent to the surface (i.e., surface area change) and expansion/compression normal to the surface, we compute a third shape descriptor called the normal Jacobian. The normal Jacobian is the ratio between the Jacobian determinant and the surface Jacobian signifies the ratio between the length of an infinitesimal line oriented normally to the surface before and after the transformation. We analyze the surface and normal log-Jacobians independently. A positive (negative) surface log-Jacobian value denotes an expansion (contraction) of the template around that vertex in the direction tangent to the surface to fit the subject. Similarly, a positive (negative) normal Jacobian value denotes an expansion (contraction) of the template around that vertex in the direction normal to the surface to fit the subject. We fit the statistical model to each vertex, thus giving $N$ models for each subject, where $N$ is the number of vertices in the population template. The null hypothesis is:

$${H}_{0}: \text{jac }= {c}_{\text{i}}\text{ICV }+ {c}_{\text{a}}\text{Age }+{c}_{\text{s}}\text{Sex }+ {c}_{0}$$

**Fig. 2:**
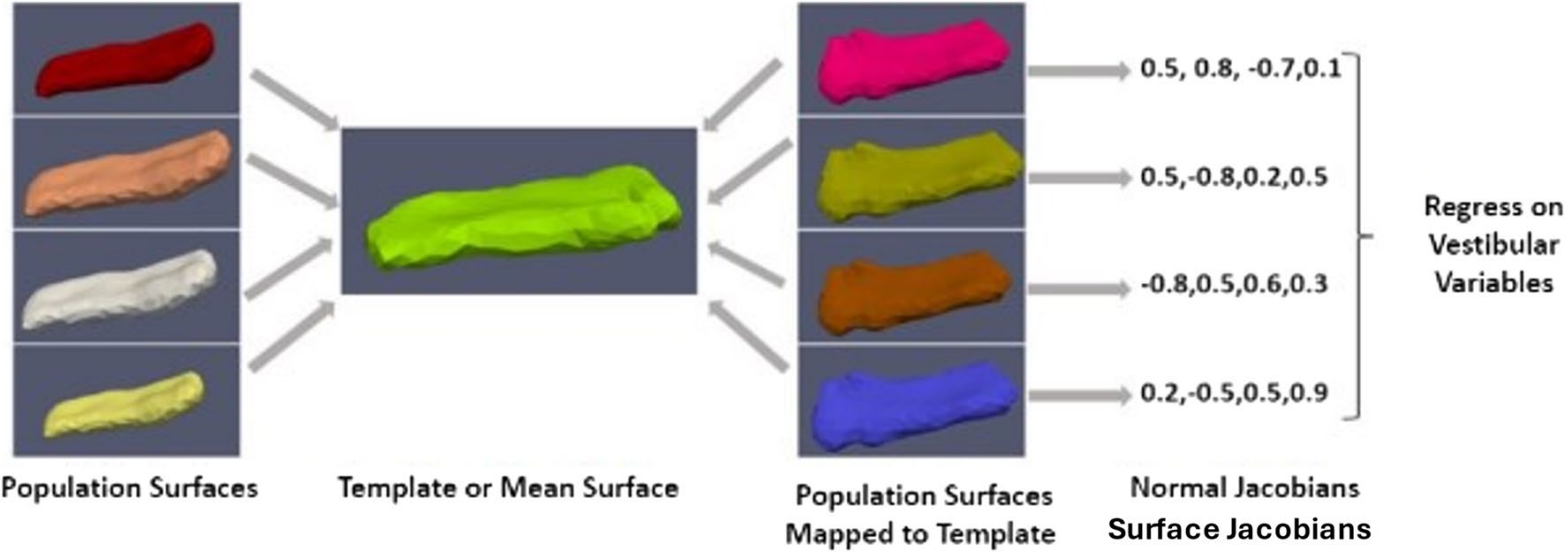
Schematic of shape analysis

The alternate hypothesis is:

$${H}_{\text{A}}:\text{ jac }= {c}_{\text{V}}\text{Vest}+{c}_{\text{i}}\text{ICV }+ {c}_{\text{a}}\text{Age }+{c}_{\text{s}}\text{Sex }+ {c}_{0}$$

where $\text{Vest}$ refers to the vestibular variables investigated (cVEMP, oVEMP, VOR Gain) and $\text{ICV}$ (intracranial volume) refers to the total volume within the skull, including left and right hemispheres, brain stem, cerebellum, and CSF (cerebral spinal fluid); therefore, $\text{ICV}$ reflects the head size. For each participant, Age in years is a continuous variable and sex is a binary variable, coded 1 for female and 0 for male. The unknown coefficients are $\{{c}_{\text{V}}, {c}_{\text{i}}, {c}_{\text{a}}, {c}_{\text{s}}\}$ for vestibular variables, and $\text{ICV}$, age, sex, and ${c}_{0}$ are the constant terms estimated using least squares. The left and right brain structures are analyzed separately. The schematic of the procedure is shown in Fig. 2.

We use spectral clustering to partition all vertices ($N$ ≈ 400) into $k$ (6 to 7) clusters to boost the analytical power and limit the number of models being evaluated simultaneously [32]. Only the shape’s surface geometry is used in this procedure. Each segment’s maximal surface Jacobian is assigned to that cluster, resulting in $k$ super-vertices [19]. In Fig. 7, each colored region represents one cluster, thus reducing the number of models from $N$ (number of vertices, ≈ 400) to $k\, a$ (number of clusters, 6 to 7). Permutation testing is used to calculate significance level or *p*-value to avoid multiple hypothesis problems (see the “Multiple Hypothesis Testing” section). The relative difference was expressed in terms of presence/absence when the categorical form of vestibular variables was used. In the case of continuous form, we report the relative difference with a 1 SD increase in cVEMP, as mentioned above. For completeness, we report our results from our investigation of the relationship between cVEMP and ERC and TEC surface Jacobians which was also investigated in a previous study of the same cohort [19].

#### Multiple Hypothesis Testing

The problem of multiple hypothesis testing arises from fitting a linear model to each cluster. The probability of making at least one type I error is significantly higher than alpha if the same procedure is applied to several hypotheses tested simultaneously, particularly when the number of hypotheses is large. Here we use permutation testing to control the family-wise error rate (FWER). Permutation tests build an empirical estimate of the distribution of the test statistic under the null hypothesis by permuting/resampling data. The test statistic from the real data is then compared to this distribution, and the *p*-value is the fraction of the population greater than the real test statistic. Here, the test statistic was chosen as a ratio of maximum square errors in the null hypothesis to maximum square error in the alternate hypothesis.

### High-Field Atlasing

**Fig. 3:**
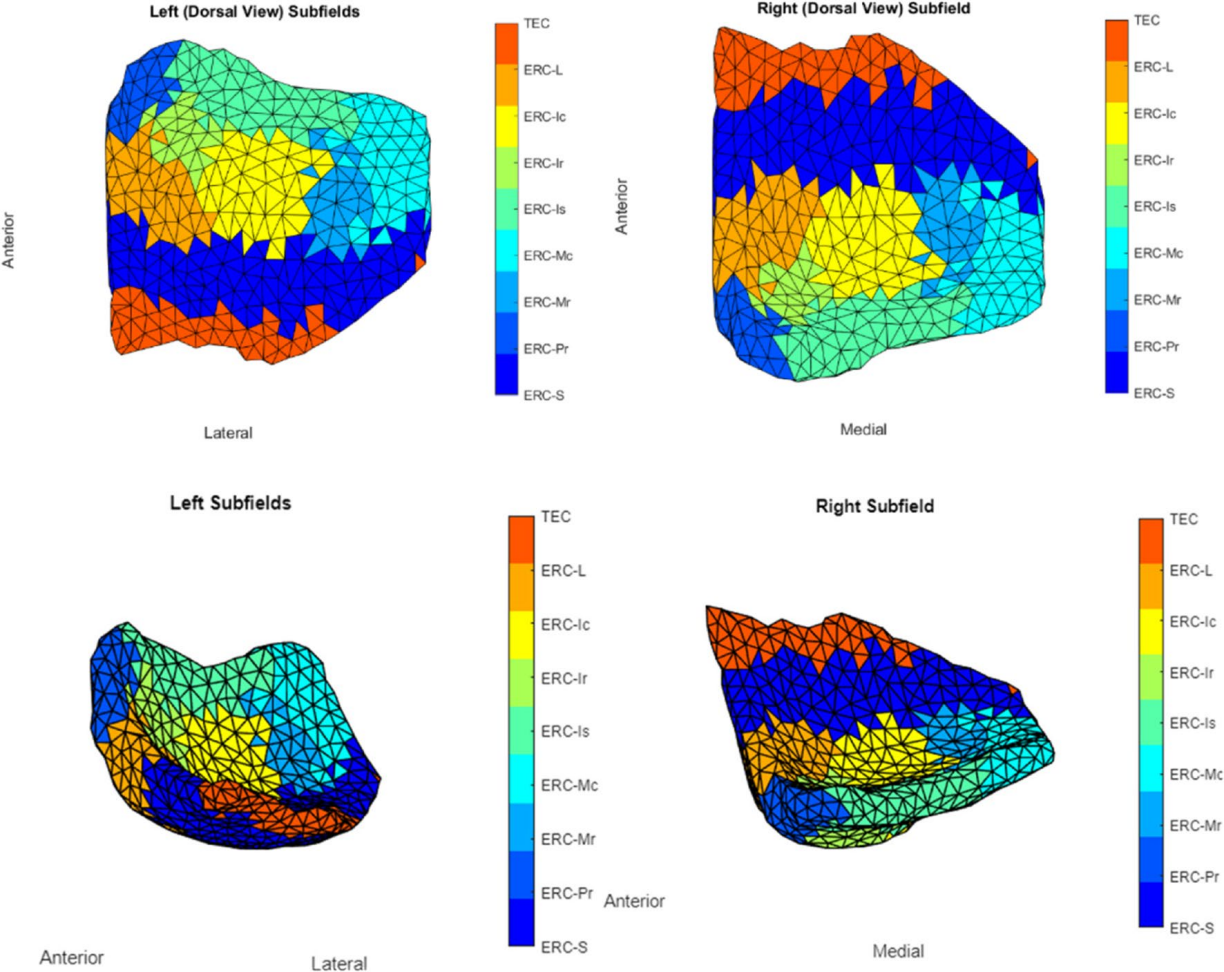
View of ERC overlayed with Krimer partitions

**Fig. 4:**
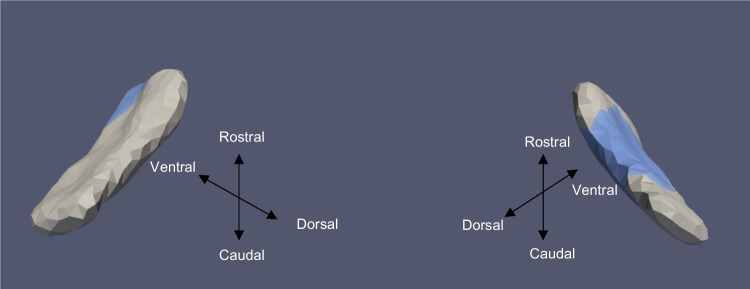
ERC template showing significant results for the clustered left and right sides of the ERC (view from the caudal end, upside-down) for categorical cVEMP

We used a high-field strength (11 T) MRI to label structures of interest on the MRI images used in this study as a posteriori. To help us define boundaries for our measurements of the ERC, we used the partitions established by Krimer et al. [33]. The Krimer partition, as it has been referred to throughout this study, divides the ERC into nine sub-regions. Established protocols were used to construct the lateral to medial coordinates for the 9 subregions [33–35]. This study defines the following 7 Krimer regions together as the ERC: (1) intermediate superior (ERC-Is), (2) intermediate rostral (ERC-Ir), (3) intermediate caudal (ERC-Ic), (4) pro-rhinal (ERC-Pr), (5) medial rostral (ERC-Mr), (6) medial caudal (ERC-Mc), and (7) lateral (ERC-L). This definition corresponds well with how the ERC is typically defined for most MRI studies. We represent the Krimer sulcal (ERC-S) and trans entorhinal regions, which correspond to the 8th and 9th Krimer atlas regions, together as the TEC [16, 18]. To assign Krimer labels to the population template, we first rigidly aligned the population template and the high-field atlas, and then we used surface LDDMM to map the population template onto the high-field atlas. Krimer labels were transferred to the population template vertices using the deformation field followed by a nearest-neighbor label assignment. Figure 3 shows the ERC population template with Krimer subfields. In a prior study, Kulason et al. (2020) highlighted the discrepancies stemming from different nomenclature about the TEC and ERC. Their comparative examination of four atlases provided insight into these disparities, with Fig. 4 in the paper as a reference point.

## Results

### Demographic Data

We had 80, 83, and 91 participants who had their cVEMP, oVEMP, and VOR measured, respectively, and MRI scans taken during the same visit. Mean cVEMP, oVEMP, and VOR amplitudes and the mean age for the cohort are shown in Table 1. Table 1 also shows the characteristics of the continuous and categorical vestibular metrics. Fifty-six percent of participants had a cVEMP response present in either ear, and 62% of participants had an oVEMP response present in either ear. Table 2 shows the percentage of continuous and discontinuous sulcus in each cohort. Several variants of continuous collateral sulcus (CoS) exist. The first variant, Type I CoS, is characterized by a deep, continuous sulcus where the rhinal sulcus shares a sulcal bed with the collateral sulcus proper [36]. This pattern has also been identified as type II/type III rhinal sulcus [37]. The second variant, termed type IIa CoS, exhibits a discontinuous CoS where the collateral sulcus proper starts posterior to the GI [36]. Additionally, the type I rhinal sulcus, aligns with type IIa CoS [37]. Finally, there is the type IIb CoS, distinguished by a discontinuous pattern where the collateral sulcus proper begins anterior to the GI [36]. Despite the differences in morphological sub-type of the collateral sulcus, we include all ERC and TEC surfaces in our analysis to maintain our sample size. Table 2 shows the groups of sulcus defined with respect to categorical vestibular measures.

**Table 1:** Characteristics of study sample (*N* = 103)

Characteristic	Mean (SD)	N (%)
Age (y)	76.99 (8.64)	
Intracranial volume (mm^3^)	1231.1 (118.38)	
Gender		
Male		74 (72%)
Female		29 (28%)
Race		
White		75 (73%)
Black		22 (21%)
Other		6 (6%)
cVEMP		
Amplitude	1.32 (0.68)	
Response present		58 (56.31%)
oVEMP		
Amplitude	14.74 (9.63)	
Response present		64 (62%)
VOR		
Gain	0.98 (0.15)	91 (88%)

**Table 2:** Collateral sulcus distribution

Variable	Continuous sulcus (%)		Discontinuous sulcus (%)	
	Left	Right	Left	Right
cVEMP	93.75	97.5	6.25	2.5
oVEMP	93.8	97.5	6	2.5
VOR	96.7	97.8	3.3	2.2

### Results of the Surface Jacobian Analysis

**Fig. 5:**
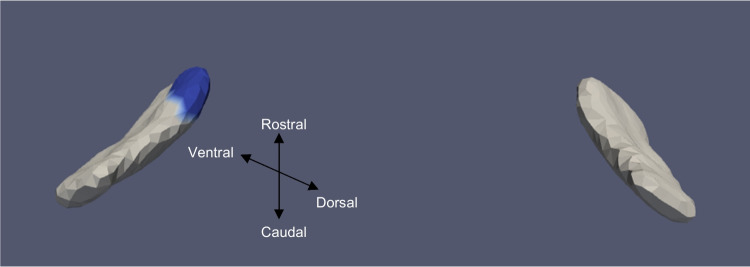
ERC template showing significant results for the clustered left and right sides of the ERC (view from the caudal end, upside-down) for categorical oVEMP

**Fig. 6:**
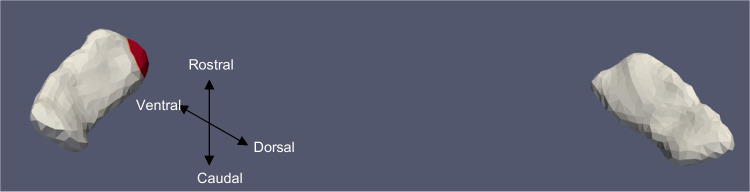
TEC template showing significant results for the clustered left and right sides of the TEC (view from the caudal end, upside-down) for continuous oVEMP

**Fig. 7:**
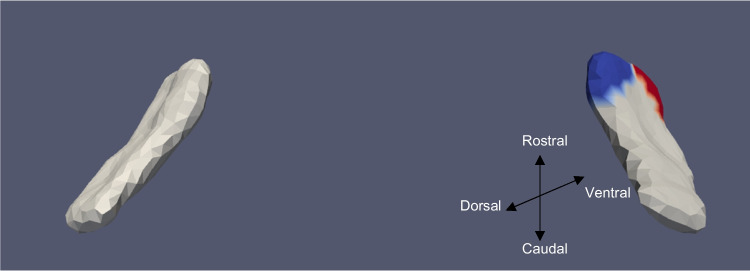
ERC template showing significant results for the clustered Left and Right sides of the ERC (view from the caudal end, upside-down) for categorical VOR

**Fig. 8:**
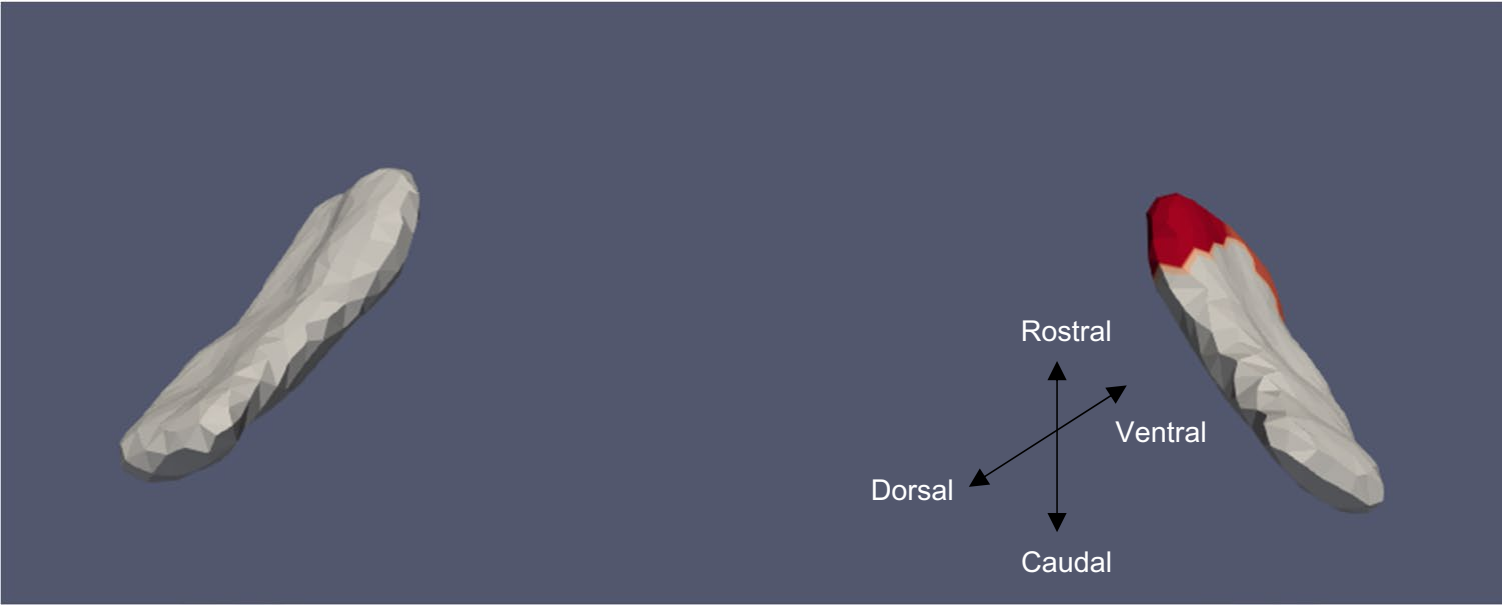
ERC template showing significant results for the clustered left and right sides of the ERC (view from the caudal end, upside-down) for continuous VOR

**Fig. 9:**
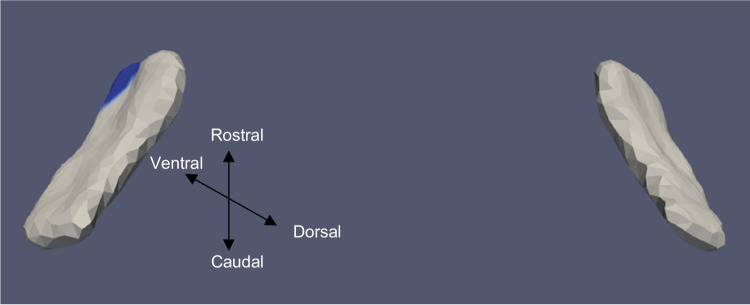
ERC template showing significant results for the clustered left and right sides of the ERC (view from the caudal end, upside-down) for categorical cVEMP

Tables 3 and 4 summarize the significant results of the surface Jacobian analysis for the ERC and the TEC, respectively. In the tables, multiple coefficients denote multiple significant clusters. Figures 4, 5, 6, 7, 8, and 9 illustrate the spatial arrangement of the vestibular effects concerning the surface Jacobian on the ERC template and TEC template, respectively. In the figures, the colors red and blue, respectively, represent expansion and compression relative to the population template.

**Table 3:** Summarizing the significant results of the surface Jacobian analysis for the ERC. A positive (negative) coefficient ${c}_{V}$ indicates surface expansion (contraction) in the direction tangent to the surface

Form of vestibular variable	Side	Coefficient (${c}_{\text{V}}$)	% change	Krimer region	p_perm
Categorical cVEMP	Left	− 0.0840	− 0.08	Pro-rhinal	0.01
	Right	− 0.108	− 0.1	Intermediate caudal and intermediate superior	0.03
Categorical oVEMP	Left	− 0.07	− 0.07	Lateral	0.03
Categorical VOR	Right	0.25, − 0.03	0.2, − 0.3	Pro-rhinal, lateral	0.01
Continuous VOR	Right	0.25. 0.27	0.25,0.27	Lateral, pro-rhinal	0.008

**Table 4:** Summarizing the significant results of the surface Jacobian analysis for the TEC. A positive (negative) coefficient ${c}_{V}$ indicates surface expansion (contraction) in the direction tangent to the surface

Form of vestibular variable	Side	Coefficient (${c}_{\text{V}}$)	% change	Krimer region	p_perm
Continuous oVEMP	Left	0.005	0.005	Lateral	0.004

The presence of cVEMP showed an association with a compression of 0.08% in the anterior medial region of left ERC (*p* ≈ 0.01), as well as with a 0.1% compression in the inferior medial region of right ERC (*p* ≈ 0.03). The TEC and cVEMP did not exhibit any significant correlation, and continuous cVEMP did not indicate any significance with the ERC. The presence of oVEMP demonstrated no link with the right side of the ERC and a substantial relationship with the left ERC (*p* ≈ 0.03) with compression of 0.07% in the anterior lateral region. Continuous oVEMP was unrelated to the ERC. The TEC did not exhibit any relationship with categorical oVEMP. One microvolt increase in oVEMP amplitude was associated with a 0.05% compression in the anterior lateral region of the left TEC (*p* ≈ 0.04). There was no relationship between the right TEC and continuous oVEMP. With an expansion of 0.25% in the anterior medial region and a compression of 0.03% in the anterior lateral region, the presence of VOR demonstrated an association with the right ERC (*p* ≈ 0.01). With an expansion of 0.2% in the anterior region, right ERC (*p* ≈ 0.008) also correlated with continuous VOR. There was no correlation between left ERC and VOR. Moreover, there was no association between the TEC and VOR gain.

### Results of the Normal Jacobian Analysis

**Fig. 10:**
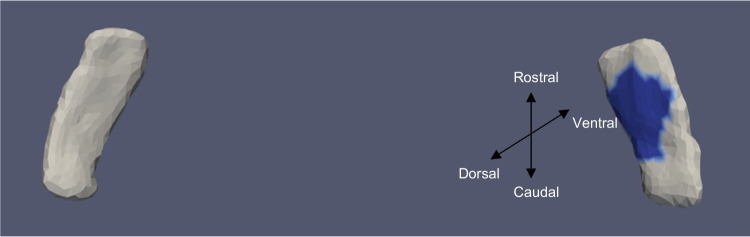
TEC template showing significant results for the clustered left and right sides of the TEC (view from the caudal end, upside-down) for categorical cVEMP

**Fig. 11:**
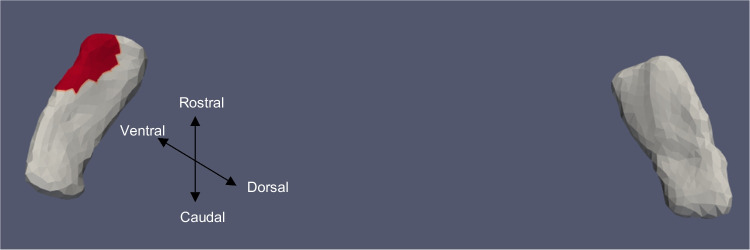
TEC template showing significant results for the clustered left and right sides of the TEC (view from the caudal end, upside-down) for continuous oVEMP

**Fig. 12:**
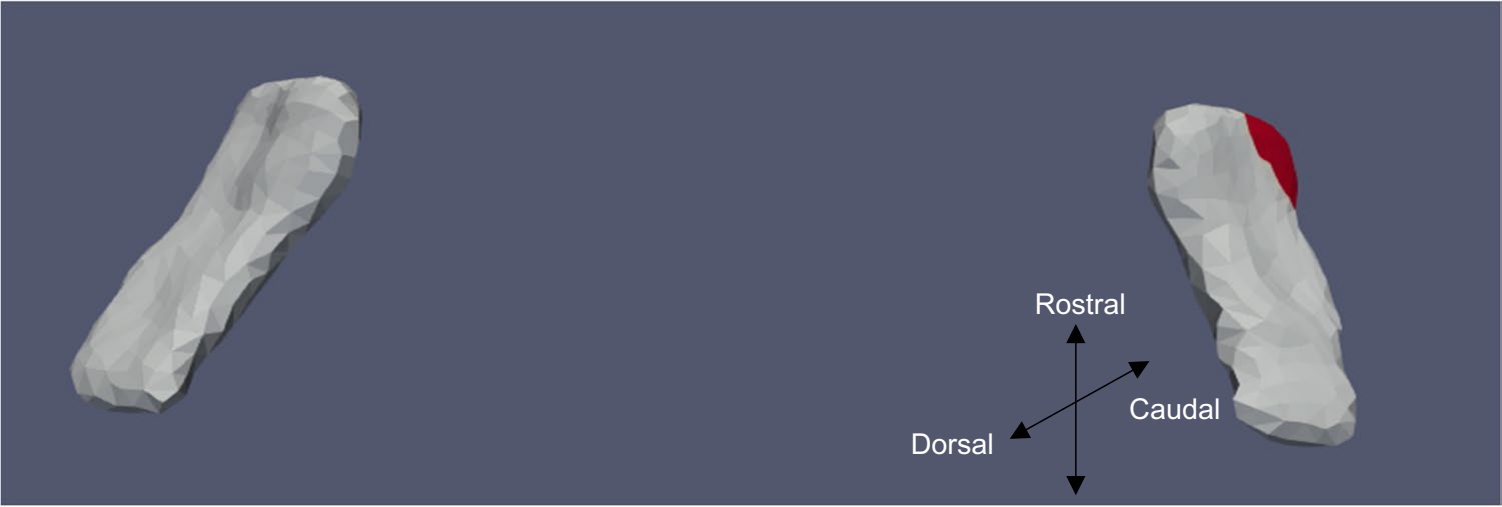
ERC template showing significant results for the clustered left and right sides of the ERC (view from the caudal end, upside-down) for categorical VOR

**Fig. 13:**
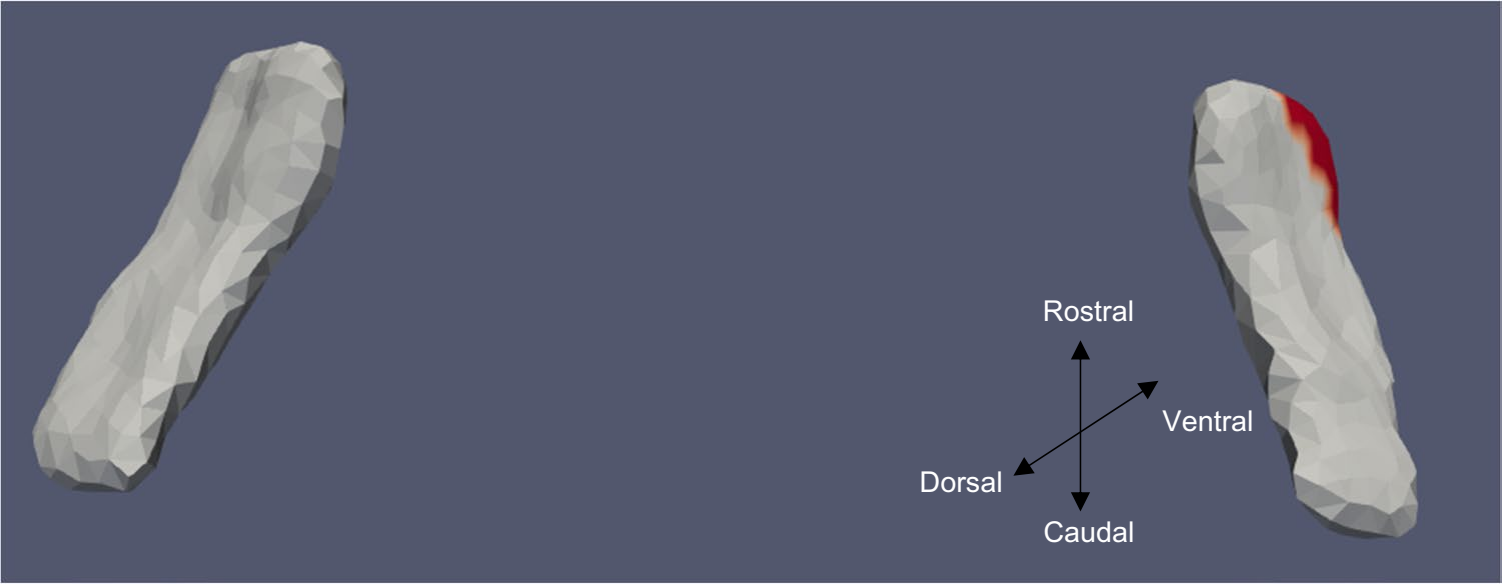
ERC template showing significant results for the clustered left and right sides of the ERC (view from the caudal end, upside-down) for continuous VOR

**Fig. 14:**
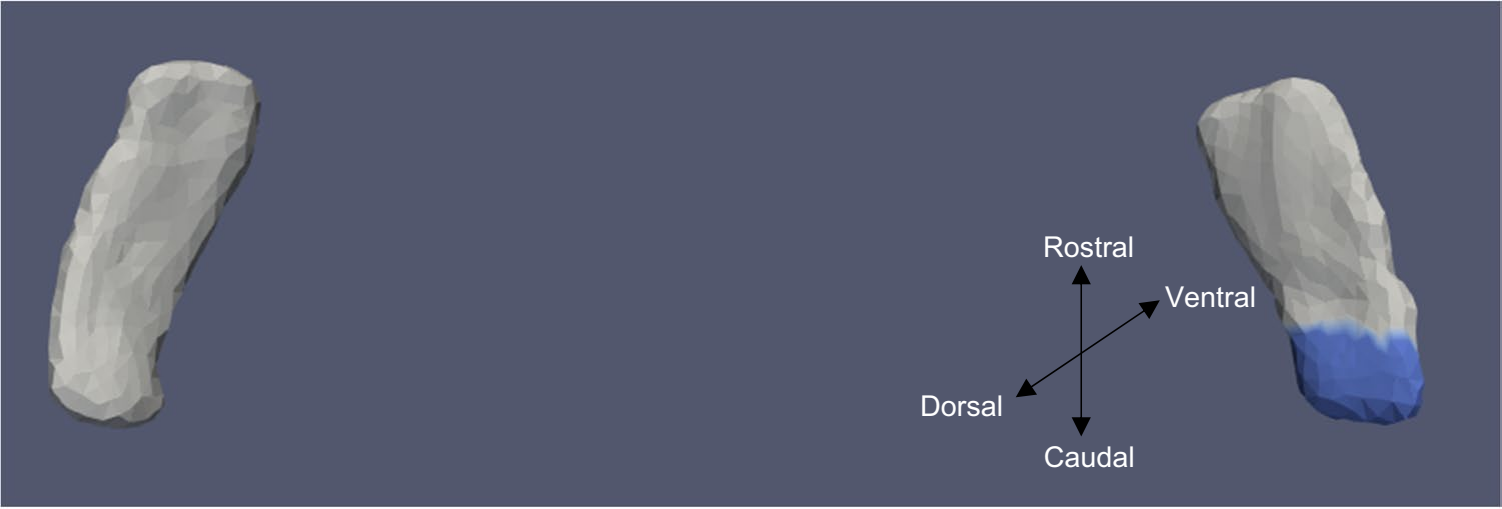
TEC template showing significant results for the clustered left and right sides of the TEC (view from the caudal end, upside-down) for categorical VOR

Tables 5 and 6 summarize the significant results of the normal Jacobian analysis for the ERC and the TEC, respectively. As in the previous section, analyses were conducted using continuous and categorical formats for all vestibular variables (cVEMP, oVEMP, and mean VOR gain). Figures 10, 11, 12, 13, and 14 illustrate the spatial arrangement of the vestibular effects concerning the normal Jacobian on the ERC template and TEC template, respectively. In the figures, the colors red and blue, respectively, represent normal surface compression or normal surface expansion relative to the mean template.

**Table 5:** Summarizing the significant results of the normal Jacobian analysis for the ERC. A positive (negative) coefficient ${c}_{\text{V}}$ indicates surface expansion (contraction) in the direction normal to the surface

Form of vestibular variable	Side	Coefficient (${c}_{\text{V}}$)	% change	Krimer Region	p_perm
Categorical—cVEMP	Left	− 0.04	− 0.04	Pro-rhinal	0.009
Categorical—VOR	Right	0.01	0.014	Pro-rhinal	0.04
Continuous—VOR	Right	0.13	0.12	Pro-rhinal	0.03

**Table 6:** Summarizing the significant results of the normal Jacobian analysis for the TEC. A positive (negative) coefficient ${c}_{\text{V}}$ indicates surface expansion (contraction) in the direction normal to the surface

Form of vestibular variable	Side	Coefficient (${c}_{\text{V}}$)	% change	Krimer region	p_perm
Categorical—cVEMP	Right	− 0.18	− 0.18	Sulcal	0.04
Continuous—oVEMP	Left	0.004	0.004	Anterior sulcal and trans-entorhinal	0.02
Categorical—VOR	Right	− 0.13	− 0.12	Posterior sulcal and trans-entorhinal	0.04

The presence of cVEMP showed an association with a compression of 0.04% in the anterior medial region of the left side of ERC (*p* ≈ 0.009) as well as with a 0.18% compression in the inferior ventral lateral region of the right side of TEC (*p* ≈ 0.04). Continuous cVEMP showed no significance with TEC or ERC. One microvolt increase in oVEMP showed significance with left TEC (*p* ≈ 0.02) with a normal expansion of 0.004% in the anterior lateral region. No significance was seen between ERC and right TEC. The presence of VOR shows an association with the right side of ERC (*p* ≈ 0.04) with a normal expansion of 0.01% in the anterior medial region. Continuous VOR shows significance with the right side of ERC (*p* ≈ 0.03) with an expansion of 0.12% also in the anterior medial region. No significance between left ERC and VOR is seen. The presence of VOR shows a significant association with the right side of TEC, with a normal compression of 0.12% (*p* ≈ 0.04) in the posterior region. No association was seen between continuous VOR and TEC.

## Discussion

In this study of healthy older adults, we analyzed the relationship between age-related vestibular end-organ loss and the shapes of the ERC and TEC—two cortical regions that putatively receive vestibular input and subserve spatial cognitive function. Our work significantly extends a previous study of the same cohort [19] which examined the relationship between saccular function as measured by cVEMP and ERC and TEC surface shape as measured by the surface Jacobian. We found cVEMP to be significantly related to the shape of the ERC, showing a relationship between reduced vestibular function and compression of the ERC. This was also reflected in the normal Jacobian relationship, which shows surface compression with reduced vestibular function. oVEMP and the shape of the ERC also displayed a similar correlation. The compressions are seen in the prorhinal, intermediate superior, and intermediate caudal Krimer regions, which correspond to the anterior medial portion of the ERC manual structural labels based on cortical folding, as shown in [16]. Furthermore, compressions were observed in the lateral Krimer region, corresponding to the anterior lateral portions of the ERC manual structural labels [16]. The lateral entorhinal cortex provides one of the two major input pathways to the hippocampus and has been suggested to process associative recognition memory [37, 38].

The dysfunction of the medial ERC results in spatial memory deficits observed in both animals and humans [39]. Moreover, it is linked with compromised spatial cognition in individuals with Alzheimer’s disease [14]. The presence of grid cells, head direction cells, speed-modulated cells, and border/boundary cells in the medial entorhinal cortex further cements the idea of it being involved in the spatial processing system [40–42]. These results align with a growing body of research in both humans and animals showing vestibular modulation of entorhinal function [10, 43–46]. Additionally, it has been demonstrated that bilateral vestibular deactivation or lesions in rats cause theta rhythm, and pattern of brain waves that are crucial for the coordination of place cells, to be disrupted in CA1 and the ERC [47–50]. Interestingly, with VOR gain, we see an expansion of the ERC. This is also reflected in the normal Jacobian relationship, which shows a positive correlation with deviation of VOR gain from normal. Specifically, the more abnormal the average VOR, the more the increase in normal Jacobian. This can be explained by the ERC’s interconnection with the hippocampus and hippocampus receiving projections from the visual centers of the brain. Jacob et al. (2020) observed an expansion in the local surface of the hippocampus with reduced vestibular function [19]. The ERC projects strongly to CA1 and CA3 of the hippocampus [51]. Signals from the semicircular canal and otolith organ converge on the medial vestibular nuclei. This vestibular information contributes to the head direction signal, which ascends to the anterior dorsal thalamus and then to the post subiculum which projects to the entorhinal cortex [11]. While both vestibular end-organs play vital roles, emerging evidence underscores the unique contributions of the otolith organs—the utricle and saccule—in spatial cognition. Unlike the semicircular canals, which detect angular acceleration, the otoliths sense linear acceleration and gravitational forces, providing a critical reference frame for spatial memory. Studies suggest that otolith dysfunction disrupts head direction cell tuning in the thalamus and destabilizes hippocampal place cells, impairing spatial memory [6, 52, 53]. These findings underscore functional distinctions between the vestibular end-organs, with the otoliths playing a particularly crucial role in processing gravitational and linear motion cues essential for spatial orientation. Although we see a connection between vestibular inputs, ERC, and hippocampus, further investigation would be required to understand why there is an increase in surface area and normal Jacobian in these regions to poorer vestibular function. Disparities have also been noted in children with ADHD, where variations in basal ganglia volume change sometimes contradicted the direction of local shape alterations [54, 55]. An increase in left and right thalamic volumes was also seen in patients with OCD [56].

For the TEC, we see a similar relationship between normal Jacobian and cVEMP as the ERC; there is a normal surface compression with reduced vestibular function. VOR and oVEMP significantly correlate with the TEC normal Jacobian and surface Jacobian, respectively, but the correlations are opposite to what we get for ERC. With VOR, we see a decrease in normal Jacobian with a more abnormal average VOR. Reduced oVEMP shows a tangential expansion in the shape of the TEC. The results of the TEC, like the ERC, can be attributed to the fact that the TEC, which contains Brodmann area 35, projects strongly to ERC [57]. Studies have also shown pathological degenerations in TEC for neurodegenerative diseases severely affecting ERC further establishing their interconnection [58]. The decrease in VOR normal Jacobian with abnormal VOR aligns with the proposition that the TEC is deemed to lie at the apex of a hierarchy of processing in the ventral visual stream [59, 60]. We observe these changes in the perirhinal cortex comprising sulcal and trans-entorhinal regions of the Krimer atlas. The perirhinal cortex plays a major role in object recognition and gives out direct projections to the lateral entorhinal cortex further emphasizing the relationship proposed in our findings.

### Limitations

A fundamental limitation of this study is that the results critically depend on the segmentation quality. Due to their blurry and ambiguous grey matter-white matter boundaries the boundary along the meninges, and their proximity to the oculomotor nerve, cortical structures like the entorhinal and trans-entorhinal cortex are notoriously challenging to segment automatically. Furthermore, the diversity of cortical shapes and structures leads to high interobserver variability, necessitating manual segmentation by an expert. The quality of manual segmentation also varies from expert to expert. Another limitation comes from spectral clustering: a strong signal region could be averaged over in a cluster to increase power, but it could also be divided into several clusters to decrease power. Additionally, using a low number of clusters reduces the resolution, restricting our ability to localize to areas smaller than a cluster size. Whether our findings can be interpreted in terms of shape or thickness, atrophy is limited. Because the surface Jacobian measuring compression and expansion reflects changes on the surface of the structure, it does not describe changes inside the structure. This limits the ability to claim whole-shape atrophy. Furthermore, the normal Jacobian differs in thickness. Whereas the normal Jacobian describes the scaling factor by which a deformed surface expanded/contracted normally to the surface at a point, thickness is defined as the lengths of the normal lines spanning two points, each on opposing surface sides (e.g., the gray-white matter surface and the gray-pial surface). A change in thickness may not imply a change in normal Jacobian, and similarly vice versa. Future histological studies would be needed to corroborate if the shape changes reported here indicate shape or thickness atrophy.

The best value from either ear is used for cVEMP and oVEMP. As a result, any side specificity is lost. This could also skew the results because if we do not account for the measurement side, we may lose any contralateral or ipsilateral dependence of shape on vestibular function. In addition, we lack knowledge of the subjects’ potential confounding variables, such as handedness and medication. As a result, the population may develop biases, which would then be reflected in the biased template. Another limitation is that the shape analysis template is based on a specific sample. The BLSA template (and resulting analyses) may not be entirely generalizable for the larger adult population to the extent that BLSA participants may differ from the general population of that same age range. Furthermore, it is unknown whether younger people with impaired vestibular function experience the same changes in brain structures that we saw in this population. Further confirmation of our analyses in other populations will be needed to confirm the robustness of our findings.

## Future Work

The relationships between age-related vestibular function and the shapes of the ERC and TEC were examined in this study. Even though a cross-sectional study of this kind helps observe relationships between various factors and structures, it does not offer conclusive details about cause-and-effect relationships. As a next step, the hypothesis that vestibular loss precedes structural atrophy can be tested in a longitudinal study. To investigate simultaneous or bidirectional relationships between vestibular function and structure, longitudinal data can be used in conjunction with statistical techniques like structural equation modeling. Additionally, changepoint analysis can be applied to longitudinal data to determine when non-linearities occur in trajectories of structural change. Furthermore, advanced analysis techniques such as network modeling can be used to evaluate how vestibular impairment in older adults may lead to a network of changes that co-occur or in characteristic sequences and how these changes relate to cognitive phenotype. These studies together may provide insights into the role of the peripheral vestibular system in spatial cognitive function in older adults.

## Supplementary Information

Below is the link to the electronic supplementary material.

Supplementary file1 (DOCX 20 KB)

## Data Availability

Data from the BLSA are available on request by proposal submission through the BLSA website (blsa.nih.gov). All requests are reviewed by the BLSA Data Sharing Proposal Review Committee and are also subject to approval from the NIH institutional review board.

## Code Availability

The codes used in this manuscript are available upon request.
