# Supplementary material for: The Inner Ear and Aging Brain: A Cross-Sectional Study of Vestibular Function and Morphometric Variations in the Entorhinal and Trans-Entorhinal Cortex
Source: J Assoc Res Otolaryngol. 2025 Feb 24;26(2):171–84. doi: 10.1007/s10162-025-00977-2 (PMC11996744; doi:10.1007/s10162-025-00977-2)
Supplement: Supplementary file 1 — Supplementary file1 (DOCX 20 KB) [file 10162_2025_977_MOESM1_ESM.docx]

## **Results of the Surface Jacobian Analysis**

Table 1: Summarizing the non-significant results of the surface Jacobian analysis for the ERC.

| **Form of Vestibular Variable** | **Side** | **p_perm** |
| --- | --- | --- |
| **Continuous cVEMP** | Left | 0.07 |
|  | Right | 0.19 |
| **Continuous oVEMP** | Left | 0.1 |
|  | Right | 0.22 |
| **Categorical VOR** | Left | 0.05 |
| **Continuous VOR** | Left | 0.1 |

Table 2:Summarizing the non-significant results of the surface Jacobian analysis for the TEC.

| **Form of Vestibular Variable** | **Side** | **p_perm** |
| --- | --- | --- |
| **Categorical cVEMP** | Left | 0.1 |
|  | Right | 0.27 |
| **Continuous cVEMP** | Left | 0.31 |
|  | Right | 0.24 |
| **Categorical oVEMP** | Left | 0.61 |
|  | Right | 0.97 |
| **Continuous oVEMP** | Right | 0.95 |
| **Categorical VOR** | Left | 0.35 |
|  | Right | 0.06 |
| **Continuous VOR** | Left | 0.96 |
|  | Right | 0.33 |

## **Results of the Normal Jacobian Analysis**

Table 3: Summarizing the non-significant results of the normal Jacobian analysis for the ERC.

| **Form of Vestibular Variable** | **Side** | **p_perm** |
| --- | --- | --- |
| **Categorical cVEMP** | Right | 0.91 |
| **Continuous cVEMP** | Left | 0.22 |
|  | Right | 0.75 |
| **Categorical oVEMP** | Left | 0.16 |
|  | Right | 0.83 |
| **Continuous oVEMP** | Left | 0.46 |
|  | Right | 0.75 |
| **Categorical VOR** | Left | 0.39 |
| **Continuous VOR** | Left | 0.11 |

Table 4: Summarizing the non-significant results of the normal Jacobian analysis for the TEC.

| **Form of Vestibular Variable** | **Side** | **p_perm** |
| --- | --- | --- |
| **Categorical cVEMP** | Left | 0.12 |
| **Continuous cVEMP** | Left | 0.44 |
|  | Right | 0.09 |
| **Categorical oVEMP** | Left | 0.39 |
|  | Right | 0.98 |
| **Continuous oVEMP** | Right | 0.96 |
| **Categorical VOR** | Left | 0.8 |
| **Continuous VOR** | Left | 0.75 |
|  | Right | 0.55 |
